# Supplementary material for: A global scoping review of the circumstances of care seeking for abortion later in pregnancy
Source: PLOS Glob Public Health. 2024 Dec 5;4(12):e0003965. doi: 10.1371/journal.pgph.0003965 (PMC11620362; doi:10.1371/journal.pgph.0003965)
Supplement: S2 Table — (DOCX) [file pgph.0003965.s002.docx]

**S2 Table.** Grey literature sources

| **Organization** | **Website** | **Citation for included report** |
| --- | --- | --- |
| Guttmacher Institute | https://www.guttmacher.org/ | Jones et al. 2019, [88] |
| Ipas | https://www.ipas.org/ | NA |
| Ibis Reproductive Health | https://www.ibisreproductivehealth.org/ | NA |
| Center for Reproductive Rights | https://reproductiverights.org/ | NA |
| Gynuity | https://gynuity.org/ | NA |
| Marie Stopes International | https://www.msichoices.org/ | NA |
