# Supplementary material for: A global scoping review of the circumstances of care seeking for abortion later in pregnancy
Source: PLOS Glob Public Health. 2024 Dec 5;4(12):e0003965. doi: 10.1371/journal.pgph.0003965 (PMC11620362; doi:10.1371/journal.pgph.0003965)
Supplement: Attachment — Submitted filename: Jacobson_response.docx [file pgph.0003965.s003.docx]

May 16, 2024

Dear Drs. Catherine Kyobutungi & Madhukar Pai Editors in Chief

The first time we submitted our manuscript our search time period was from 2007-2022. Due to internal approval processes, we were delayed in the submission of this manuscript. However, upon comments from the editorial office that this timeframe does not meet standards of “up-to-date overview of the research topic covered,” we conducted an initial search and review of manuscripts published from 2023-April of 2024. This resulted in an additional 67 articles that were reviewed by full text of abstract and 6 additional manuscripts that met inclusion criteria and were added to our methods and results sections as well as the tables and figures. The additions of these 6 papers did not change the overall conclusion or discussion of the paper, so those sections were unchanged.

We are pleased to now submit our manuscript, “A global scoping review of the circumstances of care seeking for abortion later in pregnancy” for consideration for publication in *PLOS Global Public Health*. Portions of this analysis were presented at the International Federation of Gynecology & Obstetrics (FIGO) Meeting, October 2023, Paris, France. However, the manuscript has not been publicly disclosed.

Sincerely,

Bela Ganatra, MD

UNDP/UNFPA/UNICEF/WHO/World Bank Special Programme of Research, Development & Research Training in Human Reproduction.

Department of Sexual & Reproductive Health & Research. World Health Organization Geneva, Switzerland

Laura Jacobson, PhD, MPH Consultant

UNDP/UNFPA/UNICEF/WHO/World Bank Special Programme of Research, Development & Research Training in Human Reproduction.

OHSU-PSU School of Public Health, Portland OR, USA
