# Supplementary material for: A global scoping review of the circumstances of care seeking for abortion later in pregnancy
Source: PLOS Glob Public Health. 2024 Dec 5;4(12):e0003965. doi: 10.1371/journal.pgph.0003965 (PMC11620362; doi:10.1371/journal.pgph.0003965)
Supplement: Attachment — Submitted filename: PLOS_response_to_reviewers.docx [file pgph.0003965.s004.docx]

July 23, 2024

Dear Drs. Catherine Kyobutungi & Madhukar Pai Editors in Chief and Camila Gianella Malca, PhD, Academic Editor

Thank you for the opportunity to revise and resubmit our manuscript, “A global scoping review of the circumstances of care seeking for abortion later in pregnancy” for consideration for publication in *PLOS Global Public Health*. We appreciate the insightful comments and believe they changes we made based on the feedback have strengthened out paper. Below you will find and point by point response to the reviewer comments with our responses in blue text. Thank you very much.

Regards,

Sincerely,

Bela Ganatra, MD

UNDP/UNFPA/UNICEF/WHO/World Bank Special Programme of Research, Development & Research Training in Human Reproduction.

Department of Sexual & Reproductive Health & Research. World Health Organization Geneva, Switzerland

Laura Jacobson, PhD, MPH Consultant

UNDP/UNFPA/UNICEF/WHO/World Bank Special Programme of Research, Development & Research Training in Human Reproduction.

OHSU-PSU School of Public Health, Portland OR, USA

Additional Editor Comments (if provided):

This paper addresses a critical issue that deserves more attention from researchers. The authors' efforts and the relevance of publishing research on this issue in academic journals are significant and their work is making a valuable contribution to the field.
While recognising the paper's relevance, it is also important to stress the challenges of performing a scoping review, such as the one presented in the article, of an issue highly dependent on the legal framework. The grounds for legal abortion are different in different contexts. Therefore, the internal validity of the analysis must explain how the authors take into account the legal framework of each of the countries included in the review. This is critical because what is called an administrative or bureaucratic barrier in one country could be related in another with a more restrictive ground for legal abortion, the result of the fair to criminalisation.

It is also essential to analyse, or at least consider, the legal reforms performed in the countries to guarantee or block access to safe abortion. For example, in India, while it is true that abortion has been legal since 1971, only in 2003 did the country implement a legal reform (issued a regulation) to improve access to free abortion services. In Mozambique, abortion has been legal since 2014, and in Colombia, the legal grounds for abortion have dramatically changed in recent years due to the decisions of the Constitutional Court.

Thank you for raising this important issue to consider in the explanation of our analysis.

Considering this, I ask the authors to review the article and explain how they have addressed the critical issue of the legal status and legal grounds for abortion in each of the studies included. For example, the legal grounds for abortion in the country where the study selected was performed were considered an inclusion/exclusion variable (the authors state that the research doesn’t contain a control variable), and how the legal grounds could affect the results.

We agree that legal context has important implications for the provision of abortion later in pregnancy and is relevant to our analysis. We have addressed this comment in 2 ways. First, we added a column to Table 3 that included any information on country legal status that the authors of the papers that we reviewed discussed in their paper. Given that we are documenting what is reported in the literature, we are keeping the results section focused on what the included papers present. We did not limit our search based on legal context, rather reported what was recorded in the literature. See table 3 for additional details.

Second, we have added a section to the discussion section that directly discusses country legal status and the likely impact on what we are seeing reported in the literature (which is the focus of our study). It’s likely that there will be fewer documented studies in countries with legal restrictions and that the information reported in the literature will over represent countries with more liberal laws. We have added the following to the discussion section: Lines: 125-132

“Legal restrictions on abortion significantly impede the collection of comprehensive high-quality data at the facility and population levels [101]. Individuals are unlikely to disclose their experiences due to stigma and fear of legal repercussions and indirect methods used to measure abortion incidence do not account for pregnancy duration [106]. These barriers contribute to the scarcity of population-based data and standardized measures for abortion later in pregnancy, as highlighted by the variability and lack of consistency in existing studies in this review. Addressing these data challenges and advocating for universal access to sexual and reproductive health services is important for understanding evidence gaps, enhancing the quality of care, and ensuring informed policy making that supports reproductive health and rights globally.”

It will also be helpful to explain the rationality behind the time frame, considering that the selected articles included registers from the 90s.

Thank you for this question. We limited the review to papers *published* between 2007-2024 to reflect the most recently available published studies and capture relatively recent abortion policies, technologies (medical abortion), and societal attitudes and person experiences. However, some included studies analyzed data representing a time much earlier than 2007 and therefore the time period reflected in our analysis extends quite a bit earlier than 2007. We also recognize the lag in the effect of policies and in publication of articles in the literature. We felt that 17 was enough time to capture a wide range of articles published from multiple countries without too much historical literature that may not be as relevant presently. We added a sentence in the methods section that explains this and now states: Lines 72-75

“This timeframe was selected to capture relatively recent trends in abortion policies, technologies (i.e. the advent of medical abortion), and current experiences in published studies; however, some included studies analyzed data that extended much earlier than 2007.”
